# Comparison between older adults and youth with body constitution: A cross-sectional study: Erratum

**DOI:** 10.1097/MD.0000000000048680

**Published:** 2026-04-24

**Authors:** Chih-Te Chu, Po-Yu Huang, Wen-Long Hu, Yu-Chiang Hung

The article “Comparison between older adults and youth with body constitution: A cross-sectional study”,^[1]^ which appears in Volume 105, Issue 12 of *Medicine*, was originally published with incorrect corresponding author information.

[*Correspondence: Chih-Te Chu, Department of Chinese Medicine, Kaohsiung Chang Gung Memorial Hospital, Kaohsiung 833253, Taiwan (e-mail: dinosaurtim@gmail.com*)]* has now been corrected to [*Correspondence: Yu-Chiang Hung, Department of Chinese Medicine, National Yang Ming Chiao Tung University, Taipei 112304, Taiwan (e-mail: hungyuchiang@gmail.com*)]*.

Furthermore, the Author Information [g] for Dr. Hung has now been corrected from [^g^Department of Chinese Medicine, Taipei City Hospital, Linsen, Chinese Medicine, and Kunming Branch, Datong District, Taipei, Taiwan.] to [^g^Department of Chinese Medicine, Taipei City Hospital, Linsen, Chinese Medicine, and Kunming Branch, Taipei, Taiwan.]
